# Pulse oximetry: why oxygen saturation is still not a part of standard pediatric guidelines in low-and-middle-income countries (LMICs)

**DOI:** 10.1186/s41479-023-00108-6

**Published:** 2023-02-05

**Authors:** Maheen Sheikh, Huzaifa Ahmad, Romesa Ibrahim, Imran Nisar, Fyezah Jehan

**Affiliations:** 1 Department of Pediatrics and Child Health, Aga Khan University, Karachi, 74800 Pakistan; 2 Department of Medicine, MedStar Washington Hospital Center, Washington, DC 20010 USA

**Keywords:** Pneumonia, Low-and-middle-income countries, Integrated management of childhood illnesses, Pulse oximetry, Oxygen therapy

## Abstract

**Background:**

With the high frequency of acute respiratory infections in children worldwide, particularly so in low-resource countries, the development of effective diagnostic support is crucial. While pulse oximetry has been found to be an acceptable method of hypoxemia detection, improving clinical decision making and efficient referral, many healthcare set ups in low- and middle-income countries have not been able to implement pulse oximetry into their practice.

**Main body:**

A review of past pulse oximetry implementation attempts in low- and middle-income countries proposes the barriers and potential solutions for complete integration in the healthcare systems. The addition of pulse oximetry into WHO health guidelines would prove to improve detection of respiratory distress and ensuing therapeutic measures. Incorporation is limited by the cost and unavailability of pulse oximeters, and subsequent oxygen accessibility. This restriction is compounded by the lack of trained personnel, and healthcare provider misconceptions. These hurdles can be combated by focus on low-cost devices, and cooperation at national levels for development in healthcare infrastructure, resource transport, and oxygen delivery systems.

**Conclusion:**

The implementation of pulse oximetry shows promise to improve child morbidity and mortality from pneumonia in low- and middle-income countries. Steady measures taken to improve access to pulse oximeters and oxygen supplies, along with enhanced medical provider training are encouraging steps to thorough pulse oximetry integration.

## Introduction

Acute respiratory infections (ARIs) are among the most common causes of mortality in children less than five years of age in developing countries [1]. Approximately 2.7 million pediatric deaths can be attributed to ARIs annually, 99% of which occur in developing countries [2], primarily from pneumonia [3]. Primary care facilities in low-and-middle-income countries (LMICs) primarily rely on history and signs and symptoms to guide therapy and allocate available resources appropriately. The World Health Organization (WHO) Integrated Management of Childhood Illness (IMCI) approach to childhood illness promotes the accurate identification of childhood illnesses and improves quality of care. These guidelines utilize clinical signs and symptoms (such as fast breathing and chest indrawing) and danger signs (such as cyanosis, inability to feed, and lethargy) to diagnose the severity of pneumonia and to guide treatment and referral [4].

Hypoxemia is a strong predictor of mortality in children with pneumonia. It occurs due to alveolar consolidation, which increases intrapulmonary shunting and causes ventilation-perfusion mismatching. Hypoxemia can be life-threatening because the lack of oxygen can lead to catastrophic organ dysfunction [5]. Hypoxemia is defined as arterial oxygen saturation less than 90% as measured by pulse oximetry at sea level. However, for individuals with chronic pulmonary disease or for those living at higher altitudes, a lower threshold may be recommended [6]. The WHO recommends initiating supplemental oxygen therapy when peripheral blood oxygen saturation (SpO_2_) falls below 90% [7, 8]. Approximately 13% of children with severe pneumonia develop hypoxemia (range 9–38%), corresponding to 1.86 million cases of hypoxemic pneumonia annually [7]. Thus, the burden of mortality attributed to hypoxemic pneumonia is substantial [6, 9].

The pulse oximeter is a non-invasive device used for monitoring SpO_2_. It is a painless and cost-effective method to detect those at risk of hypoxemia and dictates the need for supplemental oxygen therapy. Pulse oximeters are robust, inexpensive, and easily accessible in developed countries. There are several varieties of pulse oximeters that are commercially available. They can be categorized into desktop, wrist-worn, handheld and fingertip types [10]. They utilize a probe that is attached around the hand or foot of a child to measure the oxygen saturation. These devices can detect low oxygen saturation levels before clinical signs of hypoxemia such as cyanosis (which has variable predictive value) appear. Studies have shown that pulse oximetry improves outcomes in severe respiratory illness in higher income countries [6, 9]. As a result, pulse oximetry has been universally implemented in developed countries to evaluate cardiorespiratory function in children [11]. Pulse oximeters help healthcare workers with the timely detection of hypoxia and improve anticipatory management with antibiotics, early referral, and supplemental oxygen [12].

There is a dearth of data available from low-resource settings on the impact of oximetry on pediatric morbidity and mortality due to pneumonia. The available literature indicates that adding oximetry to current practice improves detection of severe pneumonia. According to Floyd et al., coupling oximetry with IMCI guidelines can prevent approximately 150,000 deaths per year in countries across Africa and Asia [13]. The combined use of pulse oximetry with IMCI has a sensitivity of 70–85% in accurately diagnosing childhood pneumonia. In contrast, IMCI alone has a sensitivity of 55% [13]. Systematic reviews of several studies looking at clinical signs that predict hypoxemia have concluded that no single or combination of signs and symptoms can detect hypoxemia as accurately as pulse oximetry [14]. Utilizing clinical signs and symptoms alone leads to unnecessary antibiotic prescription and low referral rates of patients with undetected hypoxemia who require hospital care [13]. A study done by Wandi et al.showed that the use of clinical signs alone miss approximately 30% of cases with hypoxemia. Additionally, the use of clinical signs alone result in the incorrect administration of oxygen to 30% of children without hypoxemia [9]. Cyanosis may not be clinically evident in moderate hypoxemia and is difficult to detect in severe anemia. Severe hypoxemia (SpO_2_ < 50%) can cause respiratory muscle fatigue leading to respiratory failure, hence warranting the use of tools that are comparatively more reliable predictors of hypoxemia [2]. Pulse oximeters can comparatively predict the severity of pneumonia more accurately [14, 15], decreasing the dependence on these clinical signs. Introducing oximetry can improve the correct identification and treatment of severe cases by around 44% [13]. Despite the evidence, pulse oximeters are infrequently implemented and rarely utilized in LMICs [16, 17].

On the LMIC front, scoping reviews from Sub-Saharan Africa have determined that oxygen delivery systems, including cylinders and concentrators, are present in between 42 to 94% of facilities [18]. Another study suggests that of healthcare setups in 8 LMICs, 46% never had oxygen available, 33% had oxygen available occasionally, and only 21% always had oxygen available, outlining the discrepancy in oxygen delivery systems [19]. In Nigeria, only 1 in 10 children with pneumonia received oxygen they required [20]. Whilst oxygen is difficult to procure, pulse oximeters are notably more scarce, with 0%-64% of facilities having pulse oximeters available [18].

Despite the magnitude of pneumonia-related morbidity and mortality among children and neonates in resource-poor settings, the introduction of pulse oximetry into LMIC screening guidelines still lags. Primary health care (PHC) clinics, especially in rural areas, constitute the first and only point of care available to patients [21]. Diagnostic support for pneumonia (radiology, rapid molecular tests for viral respiratory infections, stethoscopes, proficiency in auscultation) is not readily available at a first level PHC. Considering the advantages of pulse oximetry, pneumonia experts in LMICs have called for the integration of pulse oximetry in standard treatment algorithms [22]. Yet, after a decade long effort to create global pneumonia awareness through advocacy, pulse oximetry remains unavailable and underutilized in developing countries. This review focuses primarily on the obstacles to the implementation of pulse oximetry in LMICs.

## Barriers

Barriers to the universal implementation of pulse oximetry in LMICs can be broadly categorized as technical and human, as shown in Table 1.

**Table 1:** Barriers and Enablers for Broad Scale Pulse Oximetry Implementation in LMICs

	**Barriers**	**Enablers**
**Logistic Factors**	• Initial cost of pulse oximeters • After-sales maintenance and support (spare parts and battery) • Short operational life-span especially with rough use	• Low cost devices • Preventive and minimal maintenance • Devices that are robust under long-term and rough use • Minimal dependence on spare parts and battery replacement
	• Probes that are faulty, sensitive to dust and climate and easily damaged	• Probes more robust to wear and tear
	• Lack of facilities and other resources for management of hypoxic patient • Particularly, absence of sustainable oxygen; oxygen shortages	• Need-based approach for sustainable provision of oxygen • Explore use of more efficient O_2_ delivery systems • Ensuring adequate transport facilities and funding to ensure a steady supply of oxygen
	• The effect of altitude on the threshold of hypoxemia is confounded by differences in geography	• More outcome-based approaches to study effects of thresholds of hypoxemia on functional outcomes to help define a universally applicable threshold of hypoxemia with regards to the geographical variations
**Human Factors**	• Lack of expertise in installation and handling of equipment • Lack of understanding of the importance of waveform in pulse oximetry	• Better implementation of clinical and technical training in the use and maintenance of pulse oximeters utilizing the WHO pulse oximetry training manual
	• Lack of institutional clinical protocols related to newborn and childhood pneumonia • Absence of pulse oximeters in national and regional policies and guidelines	• Communication and cooperation necessary between the national health department and public and private sector hospitals to form simple guidelines and protocols for the use of pulse oximeters in improving the detection of hypoxemia
	• Pulse oximetry perceived to be more relevant for assessment of pulse rate in contrast to oxygen saturation • Supposed accuracy of clinical signs and symptoms in predicting hypoxemia	• More research and cost effective studies should be done in heterogeneous local settings to outline the role of pulse oximetry in reducing childhood mortality due to pneumonia in LMICs
	• False impression of low demand of pulse oximeters in the market	• Advocacy on the effective use of pulse oximetry in detecting hypoxemia and its potential role in decreasing mortality due to childhood pneumonia in LMICs

Technical barriers include the large number of oximeters required and their associated costs, the varying definitions of hypoxemia in use and the infrastructure limitations including limited oxygen availability.

### Limited oxygen availability

The absence of sustainable oxygen in LMICs is a major barrier to pulse oximetry screening implementation. Pulse oximeter screening can only improve health outcomes of children with hypoxemia if supplemental oxygen is available for treatment [9]. The WHO has included oxygen in its list of essential medicines, however, supplemental oxygen is a limited resource in healthcare facilities in low-income countries [23]. Incorporating pulse oximetry in IMCI guidelines will create a demand for utilization of health facilities and oxygen. A study done by Floyd et al. estimated that 15% of children hospitalized for pneumonia develop hypoxemia and approximately 1.5 million children with severe pneumonia require oxygen therapy [13]. The introduction of algorithms promoting the use of pulse oximeters for detection of hypoxemia, coupled with a reliable supply of supplemental oxygen, decreases the case fatality rate of severe pneumonia from 10% to 5.8% [9]. Improved oxygen delivery system establishment in Papua New Guinea determined a 35% decreased in the risk of child death due to pneumonia [24]. Yet oxygen has been either in short supply or inaccessible in many public and private sector hospitals in developing countries [25], especially in pre-pandemic times. Subjective rationing has been done to prioritize its use in emergency and surgical patients. It’s use has been scarcer still in primary health care settings, especially in rural and remote areas of LMICs.

Even in cases where hypoxemia is identified, roughly 27% to 29% of individuals are able to receive oxygen [18]. Testing of oxygen concentrators in Nigeria showed that 5% were providing medical-grade oxygen, while 48% were extruding air [26]. A lack of continuous sustainable oxygen supply renders moot the utility of pulse oximeters, as measurement of the oxygen saturation alone, in a patient with hypoxemia, will not change outcomes unless replacement oxygen is available. This has, by far, been the most compelling argument against the isolated use of pulse oximeters in low resource settings. Alternatively, it can be argued that implementation of pulse oximetry screening can uncover the scale of hypoxemia and can be used to illustrate the need for a large-scale effort to improve oxygen availability in LMICs [27]. The oxygen saturation level at which supplemental oxygen therapy should be started can be guided by data demonstrating impact on health outcomes. Although the WHO recommends a cut-off of oxygen saturation of < 90%, the effect of this threshold on health outcomes may be confounded by the underlying cause of pneumonia (viral versus bacterial) and differences in altitude and geography. A universal definition of hypoxemia may not be practical. This has been discussed at great length previously by others [27]. However, studies addressing the full scale of resource utilization or cost saving with use of pulse oximetry are lacking. Until such evidence clearly suggests increased resource utilization with no added cost benefit, the issue will remain unaddressed. More diverse real world implementation of pulse oximetry integrated into IMCI guidelines, utilizing varying populations and different hypoxia cut-offs, are needed, and are being conducted [28].

### Cost effectiveness

Since over a hundred countries utilize IMCI clinical guidelines, incorporating pulse oximetry into the guidelines would require large-scale manufacturing and distribution of pulse oximeters, like that of the WHO-ARI timers, a respiratory rate calculating device. A 2012 gap analysis of pulse oximetry by the global health organization, PATH, concluded that 77,000 pulse oximeters would be needed to implement oximetry in health care facilities such as operating rooms, postoperative recovery rooms, birthing centers, and pediatric wards. This would require maintaining a steady supply and distribution of units and batteries. The cost of maintenance of components such as probes and their replacement is also high and can be prohibitive [29]. It can therefore be projected that the cost of implementation will be high even if the price of manufacture is lowered. However, evidence suggests that utilizing pulse oximetry is cost-effective in the long run. Duke et al. in Papua New Guinea showed that the use of pulse oximeters with a sustainable oxygen supply for the treatment of severe pneumonia resulted in about $50 saved per disability-adjusted life year (DALY) [24]. This is remarkably lower than other interventions for reducing pneumonia mortality such as immunizations and antibiotics, where more supplier and market commitment is available [30]. A similar supply-and-demand commitment is required for pulse oximetry implementation.

Management of auxiliary components of oxygen therapy also pose a technical challenge to oximetry implementation. Aside from storage and maintenance of different sources of oxygen, including cylinders, concentrators or larger oxygen plants, delivery of supplemental oxygen also requires equipment like nasal prongs, tubing or face masks [6, 31]. Oxygen cylinders run out at inappropriate times and the cost of refilling and transportation is restrictive in many remote hospitals in low-resource settings [9].

Human factors posing a challenge to pulse oximetry implementation include provider misconceptions, a lack of trained personnel, and implementable policies and guidelines.

### Provider misconceptions

A significant proportion of health care providers in LMICs are unaware of the benefits provided by pulse oximetry screening and believe that there is low demand for pulse oximeters, a misconception that may hinder its implementation [22, 32]. A global survey of health care providers from 19 countries across Africa, Asia and South America indicated only 42% of providers considered pulse oximetry to be an important tool for pneumonia management. The same survey showed infrequent pulse oximeter use despite device availability among providers who consider it less important than other tools such as IMCI and chest radiography. In fact, pulse oximetry was perceived by many health care providers to be more relevant for assessment of pulse rate rather than being used as a probe for measuring oxygen saturation [32]. This misconception is a barrier and a result of low emphasis on the use of pulse oximeters in medical curricula and institutional clinical care protocols related to newborn and childhood pneumonia in LMICs. The absence of pulse oximetry in national and regional policies and guidelines (such as IMCI) for pneumonia care may promote this misconception. Additionally, health care providers should be aware of the discrepancy in pulse oximeter accuracy with use in patients with dark skin pigmentation. Studies have shown that pulse oximeters missed occult hypoxemia in three times more in Black patients than in White patients [33, 34].

### Lack of trained personnel

Pulse oximetry screening would add to the clinical burden understaffed primary health centers already deal with. Oxygen saturation is measured using an oximeter after approximately three minutes of stable observation of the child [9]. Additionally, in children, limb movement, unstable positioning, and hypothermia prolong recording time. This duration adds to the total time required for other physical assessment such as anthropometry, measuring temperature, and respiratory rate. This could be difficult in understaffed settings where disease burden is high and human resources are low. A potential danger in such a scenario is that of over-reliability on the oxygen saturation reading, and missing important clinical examination, the mainstay of IMCI diagnosis for pneumonia. Even if not so, increasing the sensitivity of pneumonia for referral will result in increased cost of referral and hospital overflow. The additive value of pulse oximetry on improving pneumonia diagnosis and mortality needs to be studied in more multicountry real world settings.

## Solutions

There are no immediate solutions to these barriers. A careful reevaluation of the existing evidence base and ongoing and future research can help to overcome many of the perceived obstacles. There is compelling evidence that hypoxemia is a bigger risk factor for pneumonia mortality than any constellation of clinical signs and symptoms [13, 15].

Extensive trials and investigations into the accuracy of these signs and symptoms were done in the past. Similarly, the world now needs to change gears to standardize pulse oximetry screening in LMICs. LMICs have access to low-cost, robust probes that are smartphone-connected [35]. These should be used in feasibility studies to evaluate the added benefit of using pulse oximetry in the management of childhood pneumonia in LMICs [36]. Very little research has been done due to the use of low-quality, malfunctioning devices and broken probes [29], hence more operational research in this area can inform the production of better and more robust models and probes. More cost-effectiveness studies are needed to assess the benefits of increasing community health care worker training and direct and indirect investment in pulse oximetry. Gaps in market dynamics need to be identified. For example, the role of innovative solutions such as advanced market commitment, like that adopted for the provision of vaccines, needs to be explored. This should consider the provision of pulse oximeters and their maintenance. A sustainable supply needs to be maintained, using models like the scale-up of respiratory rate counters and the provision of universal vaccination in LMICs. Advocacy is needed to generate funds for ensuring equitable supply. Gaps in knowledge amongst policy makers and potential end users need to be identified and appropriately addressed. The utility of pulse oximetry coupled with IMCI signs needs to be studied to understand the various issues in largescale implementation of pulse oximetry in low-resource areas.

Most importantly, ensuring presence of an adequate and continuous supply of low-cost oxygen for treatment of hypoxemia needs to go hand in hand with provision of pulse oximeters. A need-based approach to provision of oxygen, if employed, can ensure its availability and sustained supply in secondary and tertiary care hospitals, especially those that serve large cohorts and are easy to access in terms of geography and affordability.

Prior to the COVID-19 pandemic, LMICs have struggled with oxygen shortages that have been compounded with increased demand [20], even in the context of conservative oxygen therapeutic strategies [37]. The concern for insufficient oxygen delivery looms, albeit with a broader sweep of reach since the pandemic began. Studies done in Africa advocate for the use of oxygen concentrators as a cost-effective approach, the introduction of solar power, and health practitioner education [18]. Oxygen implementation strategies in Gambia and Fiji made use of cost-effective energy sources for reliable oxygen delivery [38]. At the governmental level, significant political commitment is required to identify the limitations in the health infrastructures of resource-poor countries implementing large-scale oxygen and pulse oximeter programs. Nationwide policies and guidelines should be implemented to ensure the availability of pulse oximeters at primary care points in resource poor settings, coupled with adequate oxygen access. In order to reduce the childhood mortality due to pneumonia in Ethiopia, their Federal Ministry of Health in collaboration with the team at Clinton Health Access Initiative developed a national newborn and child survival strategy in 2015. One component of this five-year strategy was pressing for a new policy on oxygen and pulse oximetry scale-up. The results from this wide-scale approach lead to functional oxygen availability from 62 to 100% in pediatric wards, and increasing the availability of pulse oximetry from 45 to 96% [39]. Adoption of pulse oximetry in Nigerian centers indicated swift implementation, with reporting on > 90% of admitted children within 6 to 12 months of commencement [40]. Alike, investigations from Malawi suggest that the application of pulse oximetry improved referral recommendations [41, 42]. Similar initiatives can be emulated by the health policy makers of other LMICs for tackling pneumonia-related burden of health care.

## Conclusion

Complementing IMCI guidelines with pulse oximetry screening has the potential to improve diagnosis, trigger early referral, and prevent substantial pneumonia mortality in low-resource settings. The challenges involved in universal pulse oximeters implementation need to be addressed in a systematic manner, to ensure that this diagnostic tool is available to everyone. Simultaneous investment is also required to strengthen existing primary, secondary and tertiary care facilities, especially with regards to oxygen availability, to ensure that those who are referred are treated appropriately.

## Data Availability

Not applicable.
